# The Gastro-protective Effect of Ginger (*Zingiber officinale* Roscoe) in *Helicobacter pylori* Positive Functional Dyspepsia

**DOI:** 10.15171/apb.2019.038

**Published:** 2019-06-01

**Authors:** Vahideh Ebrahimzadeh Attari, Mohammad Hosein Somi, Mohammad Asghari Jafarabadi, Alireza Ostadrahimi, Seyed-Yaghob Moaddab, Neda Lotfi

**Affiliations:** ^1^Maragheh University of Medical Sciences, Maragheh, Iran.; ^2^Liver and Gastrointestinal Diseases Research Center, Tabriz University of Medical Sciences, Tabriz, Iran.; ^3^Road Traffic Injury Research Center, Tabriz University of Medical Sciences, Tabriz, Iran.; ^4^Nutrition Research Center, Tabriz University of Medical Sciences, Tabriz, Iran.; ^5^Student Research Committee, Tabriz University of Medical Sciences, Tabriz, Iran.

**Keywords:** *Zingiber officinale*, Ginger, *Helicobacter pylori*, Dyspepsia, Gastric motility

## Abstract

***Purpose:*** The present study aimed to assess the effect of ginger (Zingiber officinale) powder supplementation on *Helicobacter pylori* eradication and improvement of dyspeptic symptoms in patients with *H. pylori* positive functional dyspepsia (FD).

***Methods:*** During this pilot study 15 patients with *H. pylori* positive FD received 3 g/d ginger powder as three 1-g tablets for 4-weeks. Dyspepsia symptoms were asked before and after the intervention using a questionnaire based on the Rome III criteria. *H. pylori* eradication was also assessed by a non-invasive stool antigen (HpSAg) test.

***Results:*** Ginger consumption accompanied by significant *H. pylori* eradication rate of 53.3% (P = 0.019) and the odds ratio (95% CI) was 8 (1.07 to 357.14). Moreover, our results showed significant changes in most of the dyspepsia symptoms after ginger supplementation.

***Conclusion:*** According to our findings, Z. officinale can be considered as a useful complementary therapy for FD. However, due to the small number of clinical trials in this area, further welldesigned clinical trials are needed to explicitly talk about its effectiveness especially about the eradication of *H. pylori*.

## Introduction

Functional dyspepsia (FD) is manifested by different gastrointestinal (GI) symptoms like gastric fullness, early satiety, nausea and vomiting, belching, bloating, heartburn and epigastric pain. It was previously known as non-ulcer dyspepsia or idiopathic dyspepsia.^1,2^

The pathophysiology of FD is not well understood, however, some factors like GI motor abnormalities, gastric hypersensitivity, psychosocial factors and *Helicobacter pylori* infection play important role.^2-4^

FD is a common public health problem around the world with prevalence rate of 11-30% based on the different definitions of it (Rome criteria I, II, III).^2^ Although FD is not a life threatening condition, it is accompanied by poor quality of life and experience of various medications and herbs.^5^ Since there is not yet any verified medical protocol to treat FD, the consumption of medicinal plants is increasing.

The rhizome of ginger (*Zingiber officinale* Roscoe, family Zingiberaceae) is one of the most popular medicinal plants worldwide. It has long been used as a remedy for different diseases including GI ailments like belching, bloating, vomiting, indigestion and constipation.^6,7^

There are also some scientific evidences regarding its gastro-protective effects like improving the dyspeptic symptoms.^7-11^ Moreover, based on the results of some experimental studies ginger treatment seems to inhibit *H. pylori* growth^12-16^ and prevent the gastric ulceration.^17^

However, to the best of our knowledge, the effect of ginger consumption alone (not in combination with other herbs) on *H. pylori* eradication has not yet been assessed in any clinical trial. Accordingly, the present study aimed to assess the effect of ginger powder supplementation on *H. pylori* eradication and improvement of dyspeptic symptoms in patients with *H. pylori* positive FD.

## Materials and Methods

### Study design and subjects

The present pilot study was conducted as a before-after clinical trial on patients with *H. pylori* positive FD who were referred to endoscopy unit of Imam Reza hospital (Tabriz, Iran). The exclusion criteria were age under 18 or over 65, clinically diagnosed peptic ulcer during endoscopy, negative urease test, hepatobiliary disease, irritable bowel syndrome and gastric cancer, having severe anorexia or vomiting, history of gastric surgery, taking antibiotics and nonsteroidal anti-inflammatory drugs, smoking and being hypersensitive to ginger.

**Figure 1:**
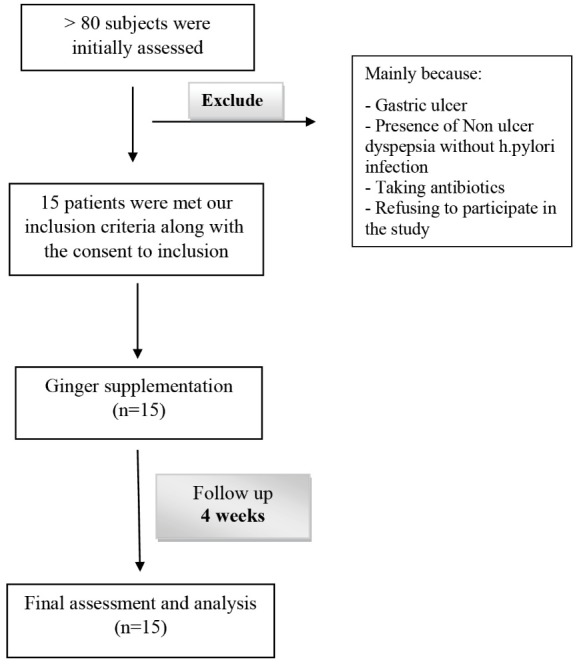
Flow diagram of study participant.

Accordingly, a total of 15 patients were recruited voluntarily of more than 80 subjects who were initially assessed (Figure 1).

### Intervention

Dried rhizomes of ginger (*Z. officinale* Roscoe, Chinese yellow ginger) were purchased from a local market in Tabriz. The ginger rhizomes were finely ground and then prepared as tablets containing 1-g ginger powder in each (Pharmaceutics laboratory, Faculty of Pharmacy, Tabriz University of Medical Science). The tablets were placed in the identical bottles. Subjects were asked to take 3 tablets per day with meals for 4 weeks.

### Assessments

Dyspepsia symptoms were asked before and after the intervention using a questionnaire based on the symptoms of Rome III criteria, including gastric fullness, early satiety, nausea, vomiting, belching, epigastric pain and heartburn which were graded through the visual analog scales (0–10 scores). Moreover, *H. pylori* eradication assessment after ginger treatment was performed by the stool antigen test (HpSAg) in an accredited laboratory since it was impossible to do the endoscopy (rapid urease test) again at the end of intervention. It should be mentioned that HpSAg has >95% sensitivity, >94% specificity, and correlation to endoscopy of 95.5%.^18^

### Statistical analysis

Data were analyzed using SPSS software, version 21.0 (IBM Corp., Armonk, NY, USA). Results were reported as mean (SD) and median (IQR). The eradication rate of *H. pylori* was assessed using the chi-square and McNemar tests. The Wilcoxon signed rank test was also used to assess the differences of GI symptoms before and after ginger supplementation. The significance level was set at *P* = 0.05.

## Results

The baseline characteristics of the participants are shown in Table 1. According to Table 2, after 4 weeks of ginger supplementation the eradication rate of *H. pylori* was 53.3 % which was statistically significant (*P *= 0.019) and the eradication rate was eight to one based on the presented odds ratio (95% CI).

**Table 1:** Basic characteristics of study subjects (n = 15)

	**Mean ± SD**
Age (y)	42.20 (16.83)
Sex, No. (%)	
Men	5 (33.3 %)
Women	10 (66.7%)
Weight (kg)	69.07 (14.35)
Height (cm)	163.47 (8.78)
BMI (kg/cm^2^)	26.04 (6.12)

**Table 2:** The eradication rate of *H. pylori* after ginger supplementation in patients with functional dyspepsia

	***H. pylori*** **positive**	***H. pylori*** **negative**	***P*** **value** ^a^	**OR (95% CI)**
Before	15	0		
After	7	8	0.019	8 (1.07 -357.14).

^a^Based on the McNemar test.

Ginger supplementation was also accompanied by some improvements in dyspepsia symptoms. In the present study, gastric fullness, nausea, belching and gastric pain were more prevalent than other symptoms in patients with FD. Regarding the non-parametric feature of dyspepsia symptoms’ scores, the median and IQR (25th, 75th percentiles) was presented for each variable in Table 3. Moreover, the mean (SD) was also reported for better understanding of changes. Based on the Wilcoxon signed rank test, there were significant changes in gastric fullness (*P *= 0.018), early satiety (*P *= 0.039), nausea (*P *= 0.018), belching (*P *= 0.016), gastric pain (*P *= 0.003) and gastric burn (*P *= 0.039) scores after ginger consumption.

**Table 3:** Comparison of the dyspepsia symptoms before and after ginger supplementation

	**Before**		**After**		***P*** **value** ^a^
	**Median (IQR)**	**Mean (SD)**	**Median (IQR)**	**Mean (SD)**	
Fullness	1 (1,7)	3.27 (2.89)	1 (1,2)	1.67 (1.95)	0.018
Early satiety	1 (1,4)	2.47 (2.58)	1 (1,1)	1.60 (1.59)	0.039
Nausea	1 (1,6)	3.13 (2.97)	1 (0,1)	1.40 (2.06)	0.018
Vomiting	1 (1,1)	1.47 (1.55)	1 (1,1)	0.87 (0.35)	0.180
Belching	2 (1,3)	2.67 (2.61)	1 (0,1)	1.27 (1.71)	0.016
Gastric pain	2 (1,4)	2.73 (1.53)	1 (0,1)	0.73 (0.88)	0.003
Gastric burn	1 (1,3)	2.40 (2.29)	1 (1,1)	1.07 (0.88)	0.039

^a^ Based on Wilcoxon signed rank test.

## Discussion

*Helicobacter pylori* infection plays important role in the pathogenesis of different gastro-duodenal diseases including gastritis, ulcers and carcinoma.^19^ Nowadays, the triple or quadruple therapy is used for the eradication of *H. pylori*. However, their eradication rate is not 100% due to resistance to antibiotics and patients’ poor compliance.^20^

According to our results, ginger supplementation caused significant *H. pylori* eradication. Ginger rhizome seems to have anti *H. pylori* activity through its different phenolic compounds (e.g., gingerol, shogaol, zingerone and phenolic acids like gallic acid and cinnamic acid).^12-15^ Results of Siddaraju and Dharmesh indicated that a hydrolysed phenolic fraction of ginger had better inhibitory effect against *H. pylori* compared to the ginger free phenolic fraction in vitro.^15^ Results of Nostro et al showed that the combination of ginger extract and clarithromycin increased the eradication rate of *H. pylori* by their synergic effects.^16^

In addition, there are some experimental and clinical reports of the anti *H. pylori* effect of multi herbal complexes including ginger.^21,22^ However, to the best of our knowledge, the anti-*H. pylori* effect of ginger alone has not yet examined in any clinical study.

The ginger extract seems to have the gastro-protective and anti-*H. pylori* effect through some mechanisms including 1) antimicrobial effect by anti-adhesive effect and also suppression of bacterial enzymes and bacterial growth; 2) inhibiting gastric acid secretion through blocking H+, K+- ATPase pomp; 3) gastro-protective effect by increased mucin secretion; 4) anti-oxidative and anti-inflammatory effects which prevent *H. pylori*-induced acute and chronic inflammation.^12,15-17^

The other finding of our study was significant changes in most of the dyspepsia symptoms after ginger supplementation which was in consistent with some previous clinical reports.^8-11^

Results of Hu et al showed that fasting consumption of 1.2 g ginger powders in patients with FD, significantly increased antral contractions and gastric emptying versus placebo.^10^

Ginger may have a modulatory effect on intestinal 5-hydroxytryptamine (5-HT; serotonin) receptors which increases GI peristalsis and decrease the food transit time.^8-11,23^

The main limitation of the present study was its low sample size. Moreover, it was better to assess the patients’ gastric infection at baseline by the stool antigen test (HpSAg) along with the rapid urease test during the endoscopy.

In conclusion, according to our findings, 3 g/d ginger powder supplementation in patients with *H. pylori* positive FD accompanied by significant eradication of *H. pylori* and improvement in dyspeptic symptoms. Therefore, *Z. officinale* can be considered as a complementary therapy for FD. However, due to the small number of clinical trials in this area, further well designed clinical trials are needed to explicitly talk about its effectiveness especially for eradication of *H. pylori*.

## Ethical Issues

The study was approved by the Ethics Committee of Tabriz University of Medical Science (reference number 9199) and registered in Iranian Registry of Clinical Trials website (identifier: IRCT201211182017N9). A written consent form was taken prior to the intervention.

## Conflict of Interest

There is no conflict of interest.

## Acknowledgments

This study was supported by a grant from Liver and Gastrointestinal Diseases Research Center, Tabriz University of Medical Sciences, Tabriz, Iran.

## References

[1] Mahadeva S, Goh KL (2006). Epidemiology of functional dyspepsia:a global perspective. World J Gastroenterol.

[2] Enck P, Azpiroz F, Boeckxstaens G (2017). Functional dyspepsia. Nat Rev Dis Primers.

[3] Vanheel H, Carbone F, Valvekens L, Simren M, Tornblom H, Vanuytsel T (2017). Pathophysiological abnormalities in functional dyspepsia subgroups according to the Rome III criteria. Am J Gastroenterol.

[4] Pribadi RR, Syam AF, Krisnuhoni E (2017). Functional dyspepsia with Helicobacter pylori Infection. Acta Med Indones.

[5] Chang L (2004). Review article: epidemiology and quality of life in functional gastrointestinal disorders. Aliment Pharmacol Ther.

[6] Ebrahimzadeh Attari V, Mahluji S, Asghari Jafarabadi M, Ostadrahimi Ostadrahimi, A A (2015). Effects of supplementation with ginger (Zingiber officinale Roscoe) on serum glucose, lipid profile and oxidative stress in obese women: a randomized, placebo-controlled clinical trial. Pharm Sci.

[7] Haniadka R, Saldanha E, Sunita V, Palatty PL, Fayad R, Baliga MS (2013). A review of the gastroprotective effects of ginger (Zingiber officinale Roscoe). Food Funct.

[8] Micklefield GH, Redeker Y, Meister V (1999). Effects of ginger on gastroduodenal motility. Int J Clin Pharmacol Ther.

[9] Wu KL, Rayner CK, Chuah SK, Changchien CS, Lu SN, Chiu YC (2008). Effects of ginger on gastric emptying and motility in healthy humans. Eur J Gastroenterol Hepatol.

[10] Hu ML, Rayner CK, Wu KL, Chuah SK, Tai WC, Chou YP (2011). Effect of ginger on gastric motility and symptoms of functional dyspepsia. World J Gastroenterol.

[11] Graeme DS (2012). Use of ginger in the management of functional dyspepsia. Gastrointest Nurs.

[12] Mahady GB, Pendland SL, Yun GS, Lu ZZ, Stoia A (2003). Ginger (Zingiber officinale Roscoe) and the gingerols inhibit the growth of CagA+ strains of Helicobacter pylori. Anticancer Res.

[13] Weseler A, Geiss HK, Saller R, Reichling J (2005). A novel colorimetric broth microdilution method to determine the minimum inhibitory concentration (MIC) of antibiotics and essential oils against Helicobacter pylori. Pharmazie.

[14] O’Mahony R, Al-Khtheeri H, Weerasekera D, Fernando N, Vaira D, Holton J (2005). Bactericidal and anti-adhesive properties of culinary and medicinal plants against Helicobacter pylori. World J Gastroenterol.

[15] Siddaraju MN, Dharmesh SM (2007). Inhibition of gastric H+, K+-ATPase and Helicobacter pylori growth by phenolic antioxidants of Zingiber officinale. Mol Nutr Food Res.

[16] Nostro A, Cellini L, Di Bartolomeo S, Cannatelli MA, Di Campli E, Procopio F (2006). Effects of combining extracts (from propolis or Zingiber officinale) with clarithromycin on Helicobacter pylori. Phytother Res.

[17] Zaman SU, Mirje MM, Ramabhimaiah S (2014). Evaluation of the anti-ulcerogenic effect of Zingiber officinale (Ginger) root in rats. Int J Curr Microbiol App Sci.

[18] Ansari S, Gautam R, Nepal HP, Subedi SN, Shrestha S, Mandal F (2016). Helicobacter pylori colonization in Nepal; assessment of prevalence and potential risk factors in a hospital-based patient cohort. ‏ BMC Res Notes.

[19] Graham DY, Yamaoka Y (1998). H pylori and cagA: relationships with gastric cancer, duodenal ulcer, and reflux esophagitis and its complications. Helicobacter.

[20] Houben MH, van de Beek D, Hensen EF, de Craen AJ, Rauws EA, Tytgat GN (1999). A systematic review of Helicobacter pylori eradication therapy--the impact of antimicrobial resistance on eradication rates‏. Aliment Pharmacol Ther.

[21] Biglar M, Sufi H, Bagherzadeh K (2014). Screening of 20 commonly used Iranian traditional medicinal plants against urease. Iran J Pharm Res.

[22] Cwikla C, Schmidt K, Matthias A, Bone KM, Lehmann R, Tiralongo E (2010). Investigations into the antibacterial activities of phytotherapeutics against Helicobacter pylori and Campylobacter jejuni. Phytother Res.

[23] Ebrahimzadeh Attari V, Malek Mahdavi A, Javadivala Z, Mahluji S, Zununi Vahed S, Ostadrahimi A (2018). A systematic review of the anti-obesity and weight lowering effect of ginger (Zingiber officinale Roscoe) and its mechanisms of action. Phytother Res.

